# Targeting Macrophage-Recruiting Chemokines as a Novel Therapeutic Strategy to Prevent the Progression of Solid Tumors

**DOI:** 10.3389/fimmu.2018.02629

**Published:** 2018-11-13

**Authors:** David Argyle, Takanori Kitamura

**Affiliations:** ^1^Royal (Dick) School of Veterinary Studies and the Roslin Institute, University of Edinburgh, Edinburgh, United Kingdom; ^2^MRC Centre for Reproductive Health, Queen's Medical Research Institute, The University of Edinburgh, Edinburgh, United Kingdom

**Keywords:** cancer, metastasis, tumor-associated macrophage, chemokine, antagonist, immunotherapy

## Abstract

Solid tumors are initiated by genetic mutations in non-hematopoietic cells and progress into invasive malignant tumors. This tumor progression often culminates in metastatic disease that is largely refractory to current therapeutic modalities and thus dramatically reduces survival of tumor patients. As solid tumors account for more than 80% of cancer-related deaths, it is necessary to develop novel therapeutic strategies to treat the diseases. An attractive strategy is to target macrophages in both primary tumors [known as tumor-associated macrophages (TAMs)] and metastatic tumors [called metastasis-associated macrophages (MAMs)]. TAMs and MAMs are abundant in most solid tumors and can promote tumor metastasis. Several studies in various models of solid tumors suggest that the accumulation of TAMs, MAMs, and their progenitor cells is regulated by chemokine ligands released by tumor and stromal cells. Consequently, these macrophage-recruiting chemokines could be potential therapeutic targets to prevent malignant tumor development through disruption of the accumulation of pro-metastatic macrophages. This review will discuss the role of chemokine ligands and their receptors in TAM and MAM accumulation in primary and secondary tumor sites, and finally discuss the therapeutic potential of inhibitors against these macrophage-recruiting chemokines.

## Introduction

Genetic alterations in non-hematopoietic cells can lead to uncontrolled cell proliferation that results in aberrant tissue mass called a solid tumor. Initially the solid tumors grow locally and do not invade adjacent tissues. However, accumulation of genetic alterations in the tumor cells turns them into malignant tumors that spread to different part of the body and establish secondary tumors (metastasis). While early detection techniques have greatly improved patient survival, significant challenges remain in the treatment of tumors following metastasis (1). It has been reported that the establishment of metastatic tumors dramatically increases the mortality rate of tumor patients (1), and thus the presence of solid tumors account for more than 80% of tumor-associated deaths (2). It is therefore necessary to prevent the metastasis formation from solid tumors.

**Figure 1:**
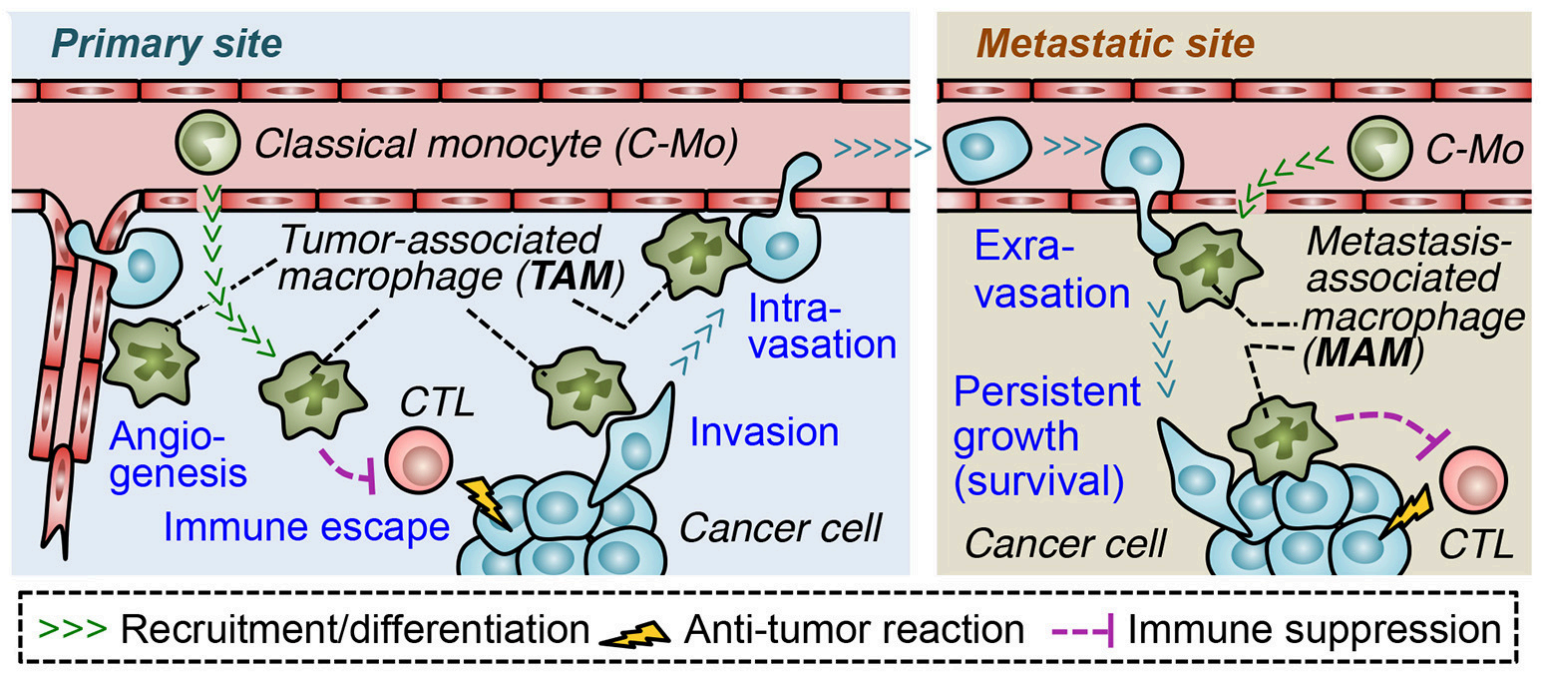
Roles of tumor-infiltrating macrophages in progression of the metastatic cascade. In the primary site, tumor-associated macrophages (TAM) suppress functions of cytotoxic lymphocytes (CTL) and promote angiogenesis, which supports tumor growth as well as dissemination of cancer cells. TAMs also directly help cancer cells to migrate into adjacent parenchyma (invasion) and to enter the blood vessels (intravasation). In the metastatic site, a distinct population of TAMs called metastasis-associated macrophage (MAM) promotes migration of cancer cells from the vessels into the parenchyma (extravasation) and their persistent growth or survival. MAMs may also suppress anti-tumor immune responses in metastatic sites. Both in the primary and secondary site, tumor-promoting macrophages (i.e., TAMs/MAMs) originate from circulating classical monocytes.

In order to form metastatic tumors, cancer cells in the solid tumors pass through a process called the metastatic cascade (3, 4). In the primary site, cancer cells escape from the anti-tumor immune responses (immune escape), invade the surrounding tissue (invasion) and enter the blood or lymphatic vessels (intravasation) that disseminate cancer cells into the circulation. The cancer cells also increase the density of blood vessels at the tumor site (angiogenesis), which also enhances tumor cell egress. At the secondary site, the circulating cancer cells migrate from the vessels to the parenchyma (extravasation) and often grow into the lethal metastatic tumors (persistent growth) (5). Through the accumulation of genetic changes, malignant tumor cells acquire several abilities that advance each step of metastasis, e.g., increased proliferation, motility, invasiveness, and survival (6). In addition to these cell autonomous changes, tumor cells require the supports from surrounding stromal cells to progress the metastatic cascade (4–6). It is now widely recognized that both primary and metastatic tumors are composed of numerous stromal cells such as endothelial cells, pericytes, fibroblasts, mesenchymal stem cells, and a variety of immune cells [including regulatory T (T_reg_) cells, mast cells, neutrophils, myeloid-derived suppressor cells (MDSCs), and tumor associated macrophages (TAMs)]. All of these stromal cells are known to promote tumor angiogenesis, cancer cell invasion, and/or disrupt immune surveillance, which helps progression of the metastatic cascade (5, 7). Among these tumor-promoting stromal cells, TAMs are one of the most abundant cell types in solid tumors (8), and a high number of TAMs in the tumor correlates with poor overall survival in most solid tumors such as breast, gastric, oral, ovarian, bladder, and thyroid cancers (9–13). Furthermore, several mouse models of malignant solid tumors have identified that TAMs recruited to primary tumors and those in the metastatic sites (called metastasis-associated macrophages, MAMs) promote almost all steps of the metastatic cascade (Figure 1) (5). Therefore, blockade of TAM and MAM accumulation in the tumor microenvironment could represent a novel approach to prevent the progression of solid tumors and improve the outcome of metastatic disease (14).

Immune cell recruitment into the site of inflammation follows several steps, i.e., tethering to the vessel wall, rolling on it, adhesion to endothelial cells, crawling, and migration through the endothelial monolayer. Since activation of certain set of integrins progress each step of this cascade, blockade of the integrin-induced adhesion cascade has been suggested as a novel therapy for inflammatory diseases (15). Another key factor that regulates the directed migration and positioning of immune cells, including macrophages, are chemokines. Chemokines are a family of small cytokines consisting of more than 50 members in human and mice. They are classified into four subfamilies based on the position of cysteine residues, i.e., XC-, CC-, CXC-, and CX3C-chemokine ligands (XCL, CCL, CXCL, and CX3CL). These chemokine ligands bind to their cognate receptors (XCR, CCR, CXCR, and CX3CR, respectively), and regulate circulation, homing, and retention of immune cells. Although some ligands can bind to multiple receptors and vice versa, the binding affinities of ligands to a cognate receptor are largely different. Furthermore, each immune cell type differentially expresses the receptors, and expression of receptors and ligands is spatially and temporally regulated (16). Therefore, each chemokine ligand-receptor pair selectively regulates the positioning of a certain type of immune cell for host defense and immunity (17). Accumulating evidences suggest that solid tumors utilize chemokines and their receptors to accomplish successful metastasis. In the tumor microenvironment, both cancer and stromal cells produce various chemokine ligands that recruit the tumor promoting immune cells such as T_reg_ cells, neutrophils, MDSCs and TAMs (18). It is therefore likely that blockade of chemokine signals could be an attractive strategy to prevent malignant tumor development by disrupting accumulation of the pro-metastatic cells including TAMs. On the other hand, the target chemokine signal should be carefully considered as it can also affect the recruitment of cytotoxic lymphocytes (CTL) such as CD8^+^ T and natural killer (NK) cells that have the potential to eliminate malignant tumor cells and thereby are essential for the success of immunotherapies such as checkpoint inhibitors and CTL transfer therapies.

In this review, I will describe the roles of TAMs and MAMs in the metastatic process, and summarize chemokine ligands and receptors that recruit the pro-metastatic macrophages mainly based on results from pre-clinical tumor models in mice. I will also discuss the therapeutic potential of TAM/MAM targeting by chemokine receptor antagonists, and consider the possibility of combining macrophage targeting with emerging immunotherapies for malignant tumors.

## Roles of macrophages in the metastatic cascade

### The contribution of TAMs to the metastatic steps at the primary site

TAMs are macrophages (characterized as F4/80^+^CD11b^+^Ly6C^low^ in mouse or CD11b^+^CD14^+^CD163^+^ in human) that accumulate in the tumor microenvironment and promote tumor progression (4). Although TAMs in solid tumors can be derived from tissue resident macrophages, several animal studies have shown that TAMs originate from classical monocytes in blood that are characterized as CD11b^+^Ly6C^+^CCR2^+^ (or CD14^++^CD16^−^CCR2^+^ in human) (14, 19). For example, a mouse model of glioblastoma has shown that adoptively transferred CCR2^+^ monocytes are recruited to the tumor and differentiate into TAMs, accounting for 85% of the total macrophage population in the tumor (20). In a mouse model of breast cancer caused by the mammary epithelial restricted expression of the Polyoma Middle T oncogene (PyMT), genetic depletion of CCR2^+^ monocytes reduces the number of TAMs in primary tumors. Further, adoptively transferred CCR2^+^ monocytes are recruited to the tumors and differentiate into F4/80^+^ macrophages (21). These results suggest that the majority of TAMs in some solid tumors are differentiated from classical monocytes.

Studies using the PyMT breast cancer model have suggested that the accumulation of TAMs promotes progression of the metastatic cascade (Figure 1). For example, macrophage ablation in the PyMT mice by genetic deletion of colony stimulating factor 1 (CSF1) suppresses tumor angiogenesis and pulmonary metastasis of cancer cells (22, 23). In this model, macrophage-selective deletion of *Wnt7b* also reduces angiogenesis in primary mammary tumors and suppresses lung metastasis (24). Tumor angiogenesis is known to promote dissemination of cancer cells from the primary tumor into the circulation by increasing the density of leaky vessels and enhancing tumor cell invasiveness (25). It is therefore likely that TAMs enhance the hematogenous dissemination of cancer cells via promoting angiogenesis. TAMs also promote the tumor cell egress by directly helping cancer cell invasion and intravasation. Intravital imaging of the PyMT tumors indicates that mammary tumor cells invade surrounding tissues together with TAMs and enter the blood vessel in association with perivascular TAMs (26, 27). In these processes, TAMs secrete epidermal growth factor (EGF), and activate its receptor in cancer cells, which enhances invasion capability and motility through increasing invadopodium formation and matrix degradation (28). It is also reported that perivascular TAMs transiently increase vascular permeability via secretion of vascular endothelial growth factor (VEGF) and thereby promote intravasation of the PyMT tumor cells (29). Consistent with these results, a high number of TAMs correlates with high density of vasculature in a variety of human solid tumors including breast cancer (30). Furthermore, direct contact between perivascular TAMs, endothelial cells and cancer cells (called tumor microenvironment for metastasis; TMEM) is associated with increased risk of distant metastasis in breast cancer (31). Several studies suggest that TAMs also protect cancer cells from anti-tumor immune reactions. For example, macrophages isolated from the mouse and human solid tumors can directly suppress T cell responses (5, 32) and NK cell cytotoxicity (33, 34) *in vitro*. It is also reported that depletion of TAMs by a CSF1 receptor antagonist enhances CD8^+^ T cell-mediated anti-tumor immunity under treatment with chemotherapy in the PyMT breast cancer mouse model (35). Mechanistically, TAMs can suppress T cell activities directly via expression of immune regulatory molecules such as arginase-1 (ARG1), IL-10, and transforming growth factor β (TGFβ) (36), as well as via physical contacts with T cells that suppresses full activation of T cells or their access to the tumor cells (37, 38). In addition, TAMs can suppress T cell-mediated immune reactions indirectly by regulating the recruitment of T_reg_ cells (39, 40). These results indicate that TAMs accumulating in primary tumors help cancer cells to disseminate into the circulation via enhancing immune suppression, angiogenesis, cancer cell motility and invasiveness. It is therefore likely that molecules that recruit TAMs can be therapeutic targets to prevent the metastatic seeding of primary tumor cells in certain types of solid tumors.

### Metastatic steps promoted by MAMs in the secondary site

It has been suggested that TAMs contain many different subtypes that play specific roles in tumor development and progression (8). In mouse models of metastatic breast cancer, a population of macrophages characterized as F4/80^high^Ly6G^−^CD11b^high^CD11c^low^ accumulates in the lung with metastatic tumors. This macrophage population is barely found in the normal lung and distinct from lung resident macrophages that are defined by high expression of F4/80 and CD11c (41, 42). The CD11b-positive macrophages that accumulate in the metastatic sites are thus called metastasis-associated macrophages (MAMs). Recent studies have shown that adoptively transferred classical monocytes are recruited to the metastatic sites where they differentiate into MAMs (43, 44). It is also reported that depletion of MAMs by CSF1 or its receptor knockout reduces metastatic tumor burden in mice that are intravenously injected with mammary tumor cells (41, 42). These results suggest that the recruitment of monocytes and subsequent accumulation of MAMs are required for circulating breast cancer cells to develop metastatic tumors.

In order to establish metastasis foci, circulating cancer cells need to extravasate, survive, and grow at the secondary sites. Several studies using mouse models of metastatic breast cancer have shown that MAMs can enhance the progression of these steps (Figure 1) (5, 8). For example, depletion of MAMs by CSF1 knockout reduces the number of cancer cells outside the blood vessels in the lung of mice that are intravenously injected with MET-1 mouse mammary tumor cells (41). It is also reported that macrophage-selective deletion of *Vegfa* reduces pulmonary metastasis formation of breast cancer cells *in vivo*, and suppresses permeability of endothelial monolayers as well as extravasation of cancer cells *in vitro* (43). These results indicate that MAMs promote extravasation of cancer cells via VEGF-A secretion. In the same model, pharmacological or genetic depletion of macrophages following tumor cell extravasation suppresses the metastatic tumor loads in the lung (41). It is also reported that MAMs suppress apoptosis of human breast cancer cells disseminated into the lung of mice by transmitting a survival signal via vascular cell adhesion molecule 1 (VCAM-1) on MDA-MB-231 human breast cancer cells (45). Furthermore, MAMs enhance angiogenesis via a Tie-2-mediated mechanism and thereby promote the outgrowth of micro-metastatic foci in the lung of PyMT mice (46). These results suggest that MAMs promote survival and persistent growth of cancer cells after seeding at the metastatic sites. Moreover, a recent study suggests that MAMs can protect cancer cells from tumoricidal immune reactions in the metastatic sites since MAMs, isolated from the metastatic tumors established by E0771-LG mouse mammary tumor cells, suppress cytotoxicity of CD8^+^ T cells against cancer cells *in vitro* (44). Given these findings, accumulation of MAMs seems to be a key factor for progression of metastatic steps at the secondary sites during pulmonary metastasis of breast cancer cells, whereas the contribution of MAMs to the development of metastasis in other tumor models or clinical patients has not yet been established.

## Chemokines that promote accumulation of pro-metastatic macrophages

### Chemokines that recruit TAMs to the primary site

**Figure 2:**
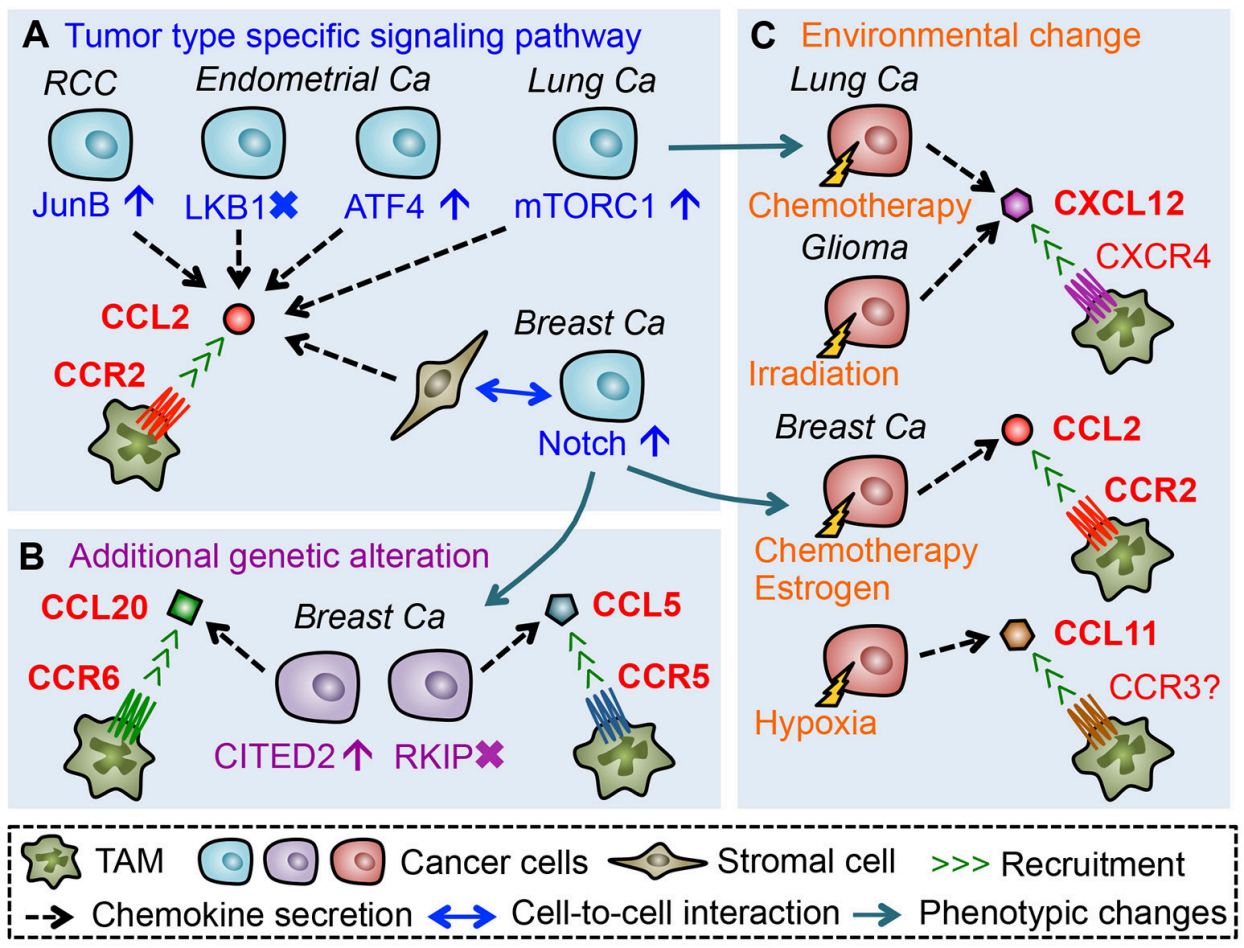
Chemokines that promote accumulation of TAMs in the primary tumors. **(A)** Tumor cells or tumor-activated stromal cells secrete CCL2 through activation or suppression of tumor type specific signaling pathways, which promote TAM accumulation in the tumor microenvironment. **(B)** Additional genetic alteration in cancer cells during tumor progression can induce expression of TAM-attracting chemokines. **(C)** Environmental changes caused by therapeutic treatments or hypoxia also promote *de novo* chemokine secretion from cancer cells.

As described above, mouse models of some solid tumors suggest that TAM accumulation in primary tumors is mainly due to the recruitment of classical monocytes that express high levels of CCR2. It is also reported that high expression of a CCR2 ligand (CCL2) in tumors positively associates with the accumulation of TAMs in glioblastoma, squamous cell carcinoma, renal cell carcinoma (RCC), as well as ovarian, endometrial, lung, and breast cancer (47–53). Thus CCL2-CCR2 signals seem to be a key determinant of monocyte recruitment and subsequent TAM accumulation. In line with this notion, several mouse studies have emphasized the importance of CCL2 in the recruitment of TAMs. For example, treatment with anti-CCL2 neutralizing antibodies significantly reduces the number of macrophages in human RCC xenografts transplanted into SCID mice, which reduces micro-vessel density, and growth of xenografted tumors (53). Although the source of CCL2 in this model is not identified, the same group has shown that a RCC cell line, 786-O, expresses high levels of CCL2. They also demonstrated that suppression of the CCL2 expression in 786-O cells reduces the number of TAMs in the xenograft tumor as well as tumor growth and microvascular density (53), suggesting that cancer cell-derived CCL2 promotes the TAM accumulation in this model (Figure 2A). In the 786-O RCC cells, the CCL2 production is increased by JunB overexpression via loss of the von Hippel-Lindau (VHL) tumor suppressor gene (54). Since loss of VHL is found in the majority of sporadic RCC and JunB is up-regulated in the VHL-deficient RCC specimens (54, 55), these results suggest that CCL2 production by cancer cells via aberrant JunB expression might be a predominant mechanism to enhance TAM accumulation in RCC. Mouse models of other types of solid tumors have also demonstrated that cancer cell-derived CCL2 plays pivotal roles in the accumulation of TAMs, whereas regulatory mechanisms behind CCL2 production differ between tumor types. For example, in subcutaneous tumors developed by LLC lung cancer cells, deletion of the *Ccl2* gene in LLC cells reduces the number of macrophages in the tumors (56). In this case, CCL2 expression in cancer cells is promoted by activation of the mammalian target of rapamycin complex 1 (mTROC1) pathway (56) that is frequently activated in various types of cancer including lung cancer (57). In endometrial cancers, established in mice by loss of liver kinase B1 (LKB1) tumor suppressor gene (*Lkb1*^−/−^), the CCL2 level is markedly increased in cancer cells. In this model, genetic deletion of *Ccl2* in the *Lkb1*^−/−^ tumors significantly reduces the number of TAMs, which results in the delayed tumor progression and prolonged overall survival (52). It is also reported that reduced expression of *LKB1* gene in immortalized human endometrial epithelial cells significantly increases CCL2 secretion (52). Consistent with these data, loss of LKB1 protein is observed in ~20% of endometrial cancers, and low LKB1 levels in the cancer strongly correlate with high CCL2 expression and high macrophage number (52, 58). Given these results, loss of LKB1 seems to be a trigger for certain populations of endometrial cancer cells to increase CCL2 expression and subsequent TAM accumulation. On the other hand, a recent study showed that AN3CA and KLE endometrial cancer cells produce CCL2 via activating transcription factor 4 (ATF4), and that anti-CCL2 neutralizing antibody treatment suppresses macrophage infiltrations in subcutaneous tumors developed by AN3CA or KLE cells (59). Since high ATF4 expression correlates with high macrophage density in human endometrial cancer (59), up-regulation of this transcription factor in cancer cells might be another mechanism behind CCL2-induced TAM accumulation in endometrial cancer. Collectively, these results suggest that cancer cells promote TAM accumulation by producing CCL2 via tumor type specific signaling pathways (Figure 2A).

The CCL2-CCR2 signaling is also required for the accumulation of TAMs and subsequent tumor progression in mouse models of breast cancer. For example, in mice with mammary tumor developed by orthotopic injection of MDA-MB-231 human breast cancer cells, treatment with anti-CCL2 antibody reduces TAM accumulation, which results in the reduced micro-vessel density and tumor growth (60). In this model, however, cancer cells may not be a major source of CCL2 since the number of TAMs does not correlate with mRNA levels of human *CCL2* in MDA-MB-231 cells but does with mouse *Ccl2* in the stroma (60). In line with this notion, immunohistochemical analysis of human breast cancer specimens shows that stromal but not tumoral CCL2 expression significantly correlates with macrophage infiltration, tumor size, and poor prognosis of patients (60). Another study also showed that genetic deletion of *Ccl2* in the host (i.e., stromal) cells but not in cancer cells results in reduced TAM infiltration, deficient angiogenesis, and impaired tumor growth in mice that are orthotopically injected with 4T1 mammary tumor cells (61). It is also reported that CCL2 is expressed in fibroblasts residing in breast cancer biopsies, and that human mesenchymal stem cells increase CCL2 secretion in response to conditioned medium from MDA-MB-231 breast cancer cells (62). Furthermore, conditioned medium from 4T1 mammary tumor cells can increase CCL2 expression in cultured macrophages (63). It is therefore likely that a population of breast cancer cells prompt stromal cells to secrete CCL2 for TAM accumulation in the tumors. Although the precise mechanism behind the stromal CCL2 production is still unclear, a recent study shows that inhibition of Notch1 expression in 4T1 cells reduces CCL2 levels in transplanted tumors and thereby decreases TAM accumulation (64). Since high Notch1 expression associates with transition from ductal carcinoma *in situ* to invasive cancer, as well as worse overall survival of breast cancer patients (65), it is possible that enhanced Notch1 expression in tumor cells during their malignant progression promotes stromal secretion of CCL2 in breast cancer (Figure 2A).

Although the above-mentioned studies suggest CCL2 as a dominant TAM attractant in most solid tumors, CCL2 inhibition suppresses TAM accumulation by only around 50% and does not achieve complete TAM depletion in the mouse models (52, 53, 56, 59–61). This suggests the involvement of other CCR2 ligands such as CCL12 (17) and cytokines such as VEGF and CSF1 that are known to recruit monocytes (66, 67). The incomplete inhibition may also be explained by the contribution of chemokine signals other than the CCL2/CCR2 axis. For example, it is reported that CCL20, a ligand for CCR6, is abundant in PyMT mammary tumors and genetic deletion of *Ccr6* gene in PyMT mice significantly reduces the number of TAMs in mammary tumors (68). Although the cell type that secretes CCL20 is unknown in the PyMT model, a recent study demonstrates that MDA-MB-231 human breast cancer cells express high level of CCL20 and that inhibition of CCL20 expression in cancer cells reduces TAM accumulation in xenografts (69). It is also reported that a highly metastatic derivative of MDA-MB-231 cells (named BM1) expresses high levels of CCL5 (a ligand for CCR5) and treatment of BM1 tumor-bearing mice with a CCR5 antagonist significantly reduces the number of TAMs in tumors (70). These results suggest that breast cancer cells can utilize CCL20-CCR6 and CCL5-CCR5 signaling in order to recruit TAMs. In the MDA-MB-231 breast cancer model (69), expression of CCL20 and TAM accumulation in xenografted tumors are suppressed by knock down of Cbp/p300-interacting transactivator with Glu/Asp-rich carboxy-terminal domain-2 (CITED2), a transcriptional co-regulator whose expression is increased in human invasive ductal carcinoma compared to normal mammary tissues and further enhanced in metastatic breast cancer (71, 72). In the BM1 as well as 4T1 mammary tumor models, forced expression of Raf kinase inhibitory protein (RKIP) suppresses CCL5 secretion from cancer cells and reduces TAM accumulation in the xenograft (70, 73). It is also reported that lower expression of RKIP in breast cancer is associated with higher levels of CCL5, as well as a higher probability of metastasis and poor prognosis (73–75). These results suggest that additional genetic alterations in cancer cells that occur in the course of tumor progression (e.g., overexpression of CITED2 and/or loss of RKIP gene) induce *de novo* chemokines (e.g., CCL20 and/or CCL5) that recruit TAMs to primary tumor sites (Figure 2B).

In addition to the genetic alterations in cancer cells, environmental changes may also switch on the *de novo* expression of TAM recruiting chemokines (Figure 2C). In mammary tumors in the PyMT mice, the CCL20-CCR6 axis can promote TAM accumulation (68) whereas the CCL2-CCR2 signal plays only a minor role if any (21). However, treatment of PyMT mice with the chemotherapeutic agent, doxorubicin, increases protein levels of CCR2 ligands CCL2 and CCL12 in the tumor stromal area, and promotes the recruitment of CCR2^+^ monocytes into the mammary tumors (76). Furthermore, in mice with the mammary tumors developed by orthotopic injection of PyMT tumor cells, treatment with estrogen enhances TAM accumulation in the tumor via increased expression of CCL2 (77). These results suggest that a chemokine signal used for the TAM accumulation in breast cancer can be switched from CCL20 to CCL2 in response to the environmental changes induced by chemotherapies or hormonal treatments. Such environmental induction of TAM recruiting chemokines can also occur locally in certain areas of the tumor. For example, a hypoxic area in a human breast cancer specimen demonstrates higher levels of CCL11 and a higher number of TAMs compared with a normoxic area (78). Since MDA-MB-231 breast cancer cells under hypoxic conditions increase CCL11 secretion and thereby promote macrophage migration *in vitro* (78), these results suggest that CCL11 is induced by low oxygen and locally recruits TAMs to the hypoxic regions in tumors. Interestingly, therapeutic treatments also promote the regional accumulation of TAMs via localized induction of CXCL12. In a mouse model of glioma, localized radiation therapy induces CXCL12 in the invasion front of xenografts, where TAMs are recruited through activation of the CXCL12 receptor, CXCR4 (79, 80). In subcutaneous tumors established by LLC lung cancer cells, chemotherapy (cyclophosphamide) treatment increases CXCL12 expression around blood vessels and recruits TAMs to the perivascular area through CXCR4 (81), whereas CCL2 from cancer cells promotes TAM accumulation in the LLC tumors without receiving any chemotherapy (82).

Taken together, it is likely that CCL2-CCR2 signaling plays a pivotal role in TAM accumulation in most solid tumors, whereas other signals such as CCL5-CCR5, CCL20-CCR6, CXCL12-CXCR4 can be an alternative or additional chemoattractant pathway (Figure 2). However, it is still unclear whether all of these chemokines are required for TAM accumulation in the same tumor microenvironment. Interestingly, a recent study using a mouse model of breast cancer showed that TAMs are recruited via CCR2 signaling to primary tumors where they induce CXCR4 expression in response to tumor-derived TGFβ and then migrate toward the blood vessel via CXCL12 to promote intravasation of cancer cells (83). It is therefore possible that TAMs utilize multiple chemokine signals for their positioning in the primary tumor in order to exert pro-metastatic functions. It is also reported that CCL2 and CXCL12 synergistically enhance the *in vitro* migration of human monocytes and macrophages (84), suggesting that expression of multiple chemokines in the tumor microenvironment is required for the efficient recruitment of monocytes and TAMs. Further investigation is necessary to identify when and how these chemokines are induced in the same tumor microenvironment and to what extent they contribute to TAM accumulation.

### Chemokines that promote MAM accumulation in the metastatic site

**Figure 3:**
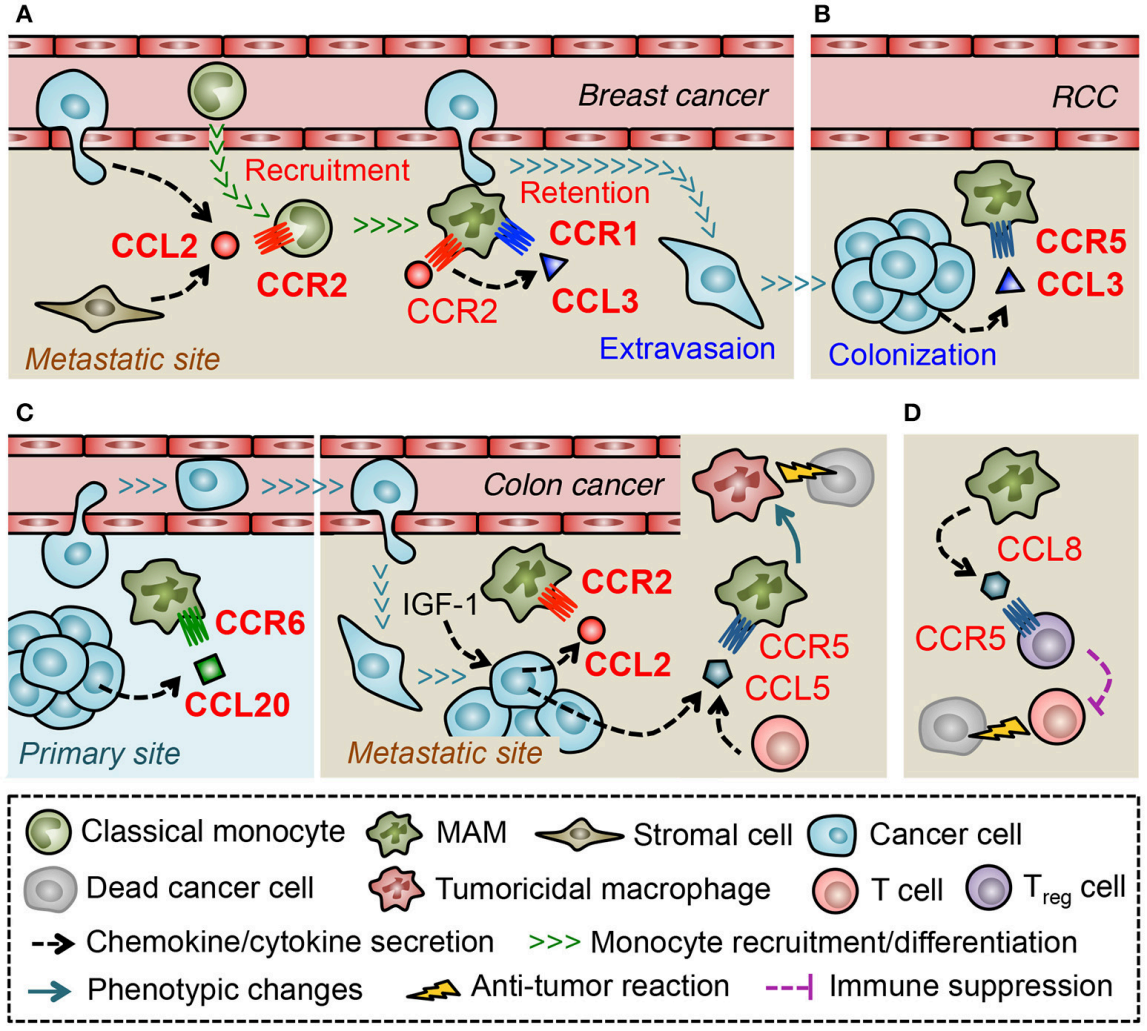
Macrophage-mediated chemokine signals in the metastatic tumors. **(A)** In the lung challenged by metastatic breast cancer cells, CCL2 recruits classical monocytes that differentiate into a distinct myeloid cell population that gives rise to metastasis-associated macrophage (MAM). Activation of CCR2 in MAMs prompts them to secrete CCL3, which in turn enhances MAM-to-cancer cell interaction and subsequent retention of MAMs via a CCR1-mediated mechanism. **(B)** In a later phase of metastasis caused by renal cell carcinoma (RCC), CCL3 in the tumor microenvironment increases MAM accumulation via a CCR5-dependent manner. **(C)** Metastasized colon cancer cells in the liver produce CCL2 via an insulin-like growth factor-1 (IGF-1) signal and recruit MAMs whereas colon cancer cells in the primary site recruit macrophages (i.e., TAMs) via CCL20 secretion. In the liver metastases of colon cancer, tumor cells, or T cells secrete CCL5 that activate CCR5 on MAMs and maintain their pro-tumor features. Thus blockade of CCR5 signal reprograms MAMs to tumoricidal cells. **(D)** In the lung metastases developed by breast cancer cells, MAMs secrete CCL8 and recruit regulatory T (T_reg_) cells.

A recent study using a mouse model of metastatic breast cancer has shown that transferred classical monocytes (F4/80^low^CD11b^+^Ly6C^+^) differentiate into MAMs (F4/80^low^CD11b^high^Ly6C^low^) by 42 h after infiltration into the lung with metastatic tumors and that the accumulation of MAMs is continuously increased during metastatic tumor growth (44). This suggests that classical monocytes are constitutively recruited and produce MAMs in metastatic tumors. It is also reported that classical monocytes expressing high levels of CCR2 preferentially migrate to metastatic tumors established by Met-1 mouse mammary tumor cells or those in the PyMT mice. In these models, anti-CCL2 antibody treatment, or genetic deletion of CCR2 inhibits the monocyte migration to the tumor-challenged lung and decreases the number of MAMs, which results in the reduction of metastatic tumor burden (43). Adoptively transferred human classical monocytes (CD14^+^CD16^−^CCR2^+^) also migrate to the metastatic tumors established by 4173 human breast cancer cells (a highly metastatic derivative from MDA-MB-231 cells) in nude mice, and this monocyte recruitment is inhibited by treatment with neutralizing antibodies against either mouse (host stromal cell-derived) or human (cancer cell-derived) CCL2 (43). Collectively, these results indicate that CCL2 secreted from both tumor cells and stromal cells plays a pivotal role in the recruitment of monocytes and subsequent accumulation of MAMs in the site of metastasis (Figure 3A).

Consistent with these results, loss of *Ccr2* significantly reduces MAM accumulation and pulmonary metastasis formation in another metastatic breast cancer model using E0771-LG mouse mammary tumor cells (42). In this model, genetic deletion of CCR1 in mice also reduces the number of MAMs in metastatic tumors and results in the decreased tumor burden. Interestingly, loss of CCR1 does not affect the recruitment of monocytes but, instead, prevents MAM-cancer cell interactions and subsequent retention of MAMs in the tumor-challenged lung (42). These results suggest that distinct chemokine signals regulate a specific process of MAM accumulation, i.e., recruitment of monocytes by CCR2 and retention of MAMs by CCR1 in pulmonary metastasis of breast cancer Figure 3A. It has been reported that freshly isolated human monocytes reduce expression of CCR2 and concomitantly increase expression of CCR1 when they differentiate to macrophages *in vitro*, and that the differentiated macrophages are more responsive to a CCR1 ligand, CCL3, than monocytes in an intracellular calcium flux assay (85). Therefore, transition of dominant receptor expression might determine differential responses of monocytes and MAMs to distinct sets of chemokine ligands. In a pulmonary metastasis model of renal cancer, MAMs increase CCR5 but not CCR1 expression at the late stage of metastatic tumor development (by 21 days after tumor injection) and loss of *Ccr5* but not *Ccr1* reduces MAM accumulation in metastatic tumors at this time point (86). On the other hand, CCR5 is not necessary for early MAM accumulation that occurs within 24 h after tumor injection in the E0771-LG breast cancer model (42). It is thus possible that the distinct microenvironments at different phases of metastasis determine predominant chemokine receptors that MAMs utilize for accumulation (Figure 3B). In line with this notion, a mouse model of liver metastasis using MC38 colon cancer cells has shown that suppression of the CCL2-CCR2 signal inhibits MAM accumulation until 9 days after intra-splenic tumor injection but fails to do so by day 13 (87).

Regulation of certain ligand expression by environmental factors may also determine the predominant chemokine signals for MAM accumulation. Although MC38 colon cancer cells release high levels of CCL2 and recruit MAMs via a CCR2-dependent manner to the liver (87), these cells produce CCL20 and recruit TAMs via a CCR6-dependent mechanism to primary tumors established by subcutaneous injection (88). Therefore, environmental factors that regulate the expression of MAM recruiting chemokines seem to be specific for the tumor site (Figure 3C). A recent study showed that treatment with an antagonist for insulin like growth factor 1 (IGF-1) receptor reduces expression of CCL2 and suppresses MAM accumulation in MC38 metastatic tumors in the liver (89), suggesting IGF-1 as a key regulator of chemokine induction in the microenvironment of tumor-challenged liver. It is notable that treatment with the IGF-1 receptor antagonist also reduces CCL5 levels in the metastatic liver (89). Although the contribution of CCL5-CCR5 signaling to MAM accumulation or the source of CCL5 was not identified in this model, a recent study using a patient-derived organotypic culture model showed that tumor-infiltrating T cells produce CCL5 (90). This study also showed that CCR5 blockade in the organotypic culture induces tumor cell death, which is abrogated by pharmacological macrophage depletion (90). This suggests that CCL5 induced by a specific tumor microenvironment prevents the MAMs to become tumoricidal cells (Figure 3C). In the E0771-LG metastatic breast cancer model, a CCR1 ligand, CCL3, is expressed by MAMs at higher level than other types of tumor-infiltrating immune cells or circulating monocytes, and loss of *Ccl3* reduces MAM accumulation in the metastatic lung. Interestingly, the CCL3 expression in MAMs is significantly suppressed by anti-CCL2 antibody treatment and recombinant CCL2 increases CCL3 secretion from cultured macrophages (42). These results collectively indicate that CCL2 in the metastatic tumor microenvironment triggers a chemokine cascade involving CCL3-CCR1 signaling that promotes retention of MAMs in the metastatic lung (Figure 3A). Since pulmonary infection with *Cryptococcus neoformans* induces CCL3 expression via a CCL2 dependent mechanism and blockade of CCL3 reduces accumulation of macrophages in the lung (91), CCL2-induced CCL3 expression may be a common mechanism for macrophage accumulation in the lung under pathological conditions. Several *in vitro* studies show such chemokine-induced chemokine production in monocytes or macrophages. For example, human monocytes cultured with CCL5 increase expression of mRNA encoding CCL2, and CCL3 (92). In human monocyte-derived macrophages, CCL18 promotes secretion of CCL2 and CCL3 as well as CCL22 that is known as a chemoattractant of T_reg_ cells (93). Interestingly, a recent report suggests that CCL3 released from E0771 breast cancer cells increases expression of CCL7, CCL8, CCL11, and CCL12 in the lung (94). Although the cell type that releases these chemokines is not clear in this study, another study using 4T1 breast cancer cells indicates that MAMs in the metastatic lung predominantly express CCL8 and recruit T_reg_ cells that express CCL8 receptor CCR5 (95) (Figure 3D). It is thus possible that distinct tumor microenvironments increase the level of chemokines such as CCL2, CCL5 and CCL18 that not only recruit monocytes/macrophages but also induce *de novo* chemokines including CCL3, CCL8, and CCL22 and thereby reinforce the accumulation of metastasis-promoting immune cells such as MAMs and T_reg_ cells (96).

Current results have indicated that spatiotemporal expression of chemokine ligands and receptors (e.g., CCL2-CCR2, CCL3-CCR1/CCR5) regulate recruitment, retention, and the phenotype of MAMs. Since these chemokine signals can be attractive targets to prevent the lethal expansion of metastatic tumors, further studies are required to understand which chemokines are expressed in a certain metastatic tumor microenvironment, how their expression is regulated, and what are their precise roles in MAM functions.

## Therapeutic potential of chemokine antagonists to prevent malignant tumor development

Different studies have identified several chemokines and chemokine receptors that promote the recruitment of TAMs into primary tumors. These chemokine ligands and receptors are potential targets to prevent dissemination of cancer cells from the primary tumors to the circulation. However, since a substantial proportion of patients (4–61% depending on the tumor sites) has already developed metastatic tumors at diagnosis, and their survival rate is < 20% in many cases (1), it is possibly more important to consider blocking the metastatic tumor outgrowth in secondary sites rather than dissemination from the primary site if we are going to improve the outcome of cancer patients. As discussed above, the CCL2-CCR2, CCL3-CCR1, and/or CCL3-CCR5 axes enhance MAM accumulation in the metastatic site, especially the lung, in mouse models of metastatic tumors. In these models, blockade of MAM accumulation via genetic deletion of CCR1, CCR2 or CCR5 significantly reduced metastatic tumor burden (43, 42, 86), suggesting that antagonists for these receptors can be novel therapeutic agents to prevent metastatic tumor development through inhibition of MAM accumulation.

CCR1 and CCR2 are well-known key regulators of immune cell accumulation, and thus several pharmaceutical companies have developed monoclonal antibodies and small molecule inhibitors against the chemokine receptors for human autoimmune diseases such as rheumatoid arthritis and multiple sclerosis (97). CCR5 antagonists have also been extensively explored since this receptor is known as a co-receptor for human immunodeficiency virus (HIV-1) to enter the cell. Consequently, the US Food and Drug Administration (FDA) has approved some CCR5 antagonists as anti-retroviral agents for HIV (97). Although these chemokine receptor antagonists were originally designed for autoimmune and infectious diseases, several pre-clinical studies have indicated their therapeutic potential for metastatic tumors. For example, a CCR1 antagonist (BL5923) can suppress metastatic tumor growth of colon cancer cells in the liver (98), and another CCR1 antagonist (CCX721) reduces tumor burden and osteolysis in a mouse model of multiple myeloma bone disease (99). In mice that have received the subcutaneous injection of LLC cancer cells, treatment with a CCR2 antagonist (RS504393) inhibits the establishment of lung metastatic foci (100). A recent study also showed that another CCR2 antagonist (RS102896) can suppress liver metastasis of MCF-7 human breast cancer cells induced by estrogen (101). Furthermore, in mice that have developed orthotopic tumors by 4T1 mammary tumor cells, treatment with a CCR5 antagonist (maraviroc) reduces metastatic tumor burden in the lung (95). Although clinical trials of chemokine receptor antagonists in cancer are still limited, several positive results have been reported. For example, an anti-CCR2 antibody (MLN1202) has been tested in a phase II clinical trial for metastatic cancer and showed therapeutic effects in 14 out of 43 patients with bone metastases (ClinicalTrials.gov ID: NCT01015560). A phase I trial of a small molecule inhibitor of CCR2 (CCX872) in combination with chemotherapy (FOLFIRINOX regimen) has also been performed in patients with non-resectable pancreatic cancer (ClinicalTrials.gov ID: NCT02345408) in which overall survival (OS) at 18 months was 29% for CCX872/FOLFIRINOX combination therapy, whereas it was 18.6% for FOLFIRINOX alone (102). A small-scale phase I clinical trial of a CCR5 antagonist (Maraviroc) in patients with metastatic colorectal cancer (ClinicalTrials.gov ID: NCT01736813) has demonstrated that maraviroc treatment in combination with chemotherapy showed an objective partial responses in three out of five patients and prolonged overall survival (90).

Despite these encouraging results, a treatment with single chemokine antagonist will not be enough to suppress metastatic tumor growth since even total deletion of CCR1, CCR2, or CCR5 by knockout cannot achieve complete elimination of metastatic tumors in mouse models (42, 86). One possible reason for this is that multiple chemokine receptors support the accumulation of pro-metastatic macrophages (i.e., TAMs and MAMs). It has been reported that solid tumors express several different chemokine ligands. For example, human colorectal cancer specimens concomitantly express CCL2, CCL4, CXCL1, CXCL5, and CXCL8 at a significantly higher level than normal mucosa (103). Further, human breast cancer tissues can express high levels of CCL2 and CCL5 compared to the adjacent normal breast tissues (77, 104). Human ovarian cancer also expresses high levels of mRNA coding CCL2, CCL4, CCL5, CXCL10, CXCL12, and CXCL16 (105). As discussed above, some receptors for these chemokines such as CCR1, CCR2, CCR3, CCR5, and CXCR4 are reported to enhance the recruitment or retention of pro-metastatic macrophages. Interestingly, several *in vitro* studies suggest that CCR1- or CCR2-induced monocyte migration is synergistically enhanced by activation of CXCR4 (84, 106), suggesting that CCR1 and CCR2 cooperate with other receptors such as CXCR4 in order to promote MAM accumulation and subsequent metastatic tumor growth. Collectively, it is possible that MAMs utilize multiple chemokine signals to accumulate in the tumor microenvironment, which makes it difficult to exclude MAMs from metastatic sites by a single chemokine receptor blockade. Therefore, inhibition of multiple chemokine receptors will be required to exercise full therapeutic effects on the MAM-promoting metastatic tumor development. An attractive approach is a treatment with dual-antagonists that inhibit more than one chemokine receptor. So far, several companies have developed dual-antagonists targeting CCR1/CCR3, CCR2/CXCR2, CCR2/CCR5, and CXCR1/CXCR2, and tested their therapeutic effects in inflammatory diseases (96, 107). For example, in genetically engineered mice that develop muscular dystrophy, treatment with a CCR2/CCR5 dual antagonist cenicriviroc reduces macrophage accumulation in the dystrophic diaphragm and slows the progression of the disease (108). In mouse models of non-alcoholic steatohepatitis (NASH), treatment with cenicriviroc reduces macrophage recruitment and ameliorates hepatic inflammation as well as fibrosis in the liver (109, 110). Since cenicriviroc treatment is well tolerated in patients with hepatic impairment without any obvious side effects (111), a phase II trial has been on going in patients with NASH and liver fibrosis (ClinicalTrials.gov ID: NCT03059446). Although clinical trials in cancer patients have not yet been reported, a mouse model where MC38 colon cancer cells were grown intramuscularly has shown that the cenicriviroc treatment can suppress TAM accumulation in the tumor and enhance therapeutic efficacy of local irradiation in suppressing tumor growth (112). These reports suggest that dual chemokine receptor antagonists are attractive drugs for cancer treatment. However, clinical application of dual-antagonists for metastatic diseases requires further identification of chemokine signal combinations that concomitantly promote MAM accumulation in metastatic tumors under different condition (e.g., tumor origin, metastatic site, and progression stage). In addition to proper target receptor selection, it is also important to determine the functional doses of antagonists that are sufficient to provide the continuous receptor coverage *in vivo* (15, 113).

**Figure 4:**
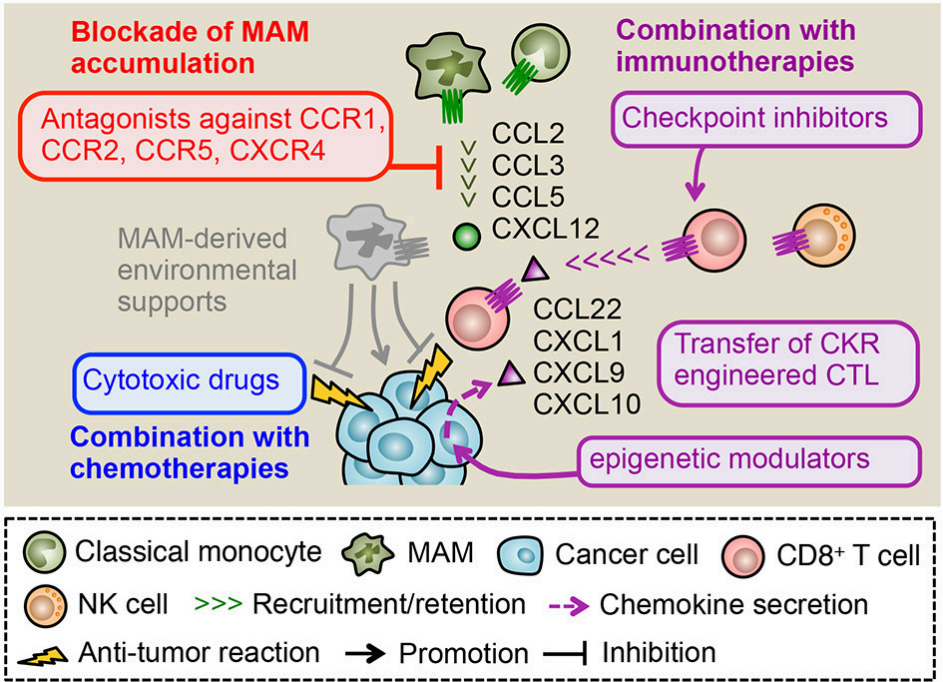
Therapeutic potential of chemokine receptor antagonists. In metastatic tumors, CCL2, CCL3, and CCL5 promote the MAM accumulation and subsequent metastasis formation. Primary tumor models also indicate a pivotal role of CXCL12 in the accumulation of macrophages whereas its role in the metastatic tumor needs to be clarified. Antagonists against receptors for these ligands (i.e., CCR1, CCR2, CCR5, and CXCR4) can reduce the number of MAMs and thus dislodge MAM-derived environmental supports for metastasized cancer cells. Several studies indicate that the MAM targeting by chemokine receptor antagonists synergistically enhance therapeutic effects of chemotherapy and immunotherapy such as immune checkpoint inhibitors and adoptive transfer of cytotoxic lymphocytes (CTL). The tumor microenvironment includes other chemokines such as CXCL9 and CXCL10 that recruit CTLs to the tumor. In some cases, epigenetic modulators can prompt cancer cells to produce these chemokines, which may enhance efficacy of MAM targeting combined with immunotherapies. Genetic manipulation of CTLs to express chemokine receptors for ligands abundantly included in tumors (e.g., CCL22, CXCL1) can also improve the efficacy of MAM targeting combined with CTL transfer therapy.

Insufficiency of a single chemokine blockade in metastasis suppression can also be due to a lack of direct cytotoxic effects on cancer cells. As described above, MAMs recruited to the metastatic site can promote tumor cell survival (41). It is also reported that malignant tumor cells express chemokine receptors such as CCR7, CXCR1, and CXCR4 that can increase their invasiveness as well as survival (114). However, it is unlikely that blockade of MAM accumulation by chemokine antagonists can directly induce tumor cell death, and thus macrophage targeting should be combined with another therapeutic modality such as chemotherapy and/or immunotherapy that directly kills the cancer cells. In line with this notion, several animal studies show that blockade of myeloid cell accumulation via chemokine receptor inhibition exerts synergistic therapeutic effects when combined with cytotoxic drug treatments. For example, reduced monocyte accumulation by genetic deletion of host CCR2 expression enhances the effect of doxorubicin or cisplatin treatment on the relapse of mammary tumors in the PyMT mice (76). Furthermore, a CXCR4 antagonist (AMD3100) prevents macrophage accumulation and delays tumor relapse after cyclophosphamide treatment in subcutaneously transplanted lung cancer and in orthotopic mammary cancers (81). In primary tumors developed by orthotopically injected pancreatic cancer cells, reduced macrophage accumulation by a CCR2 antagonist (PF-04136309) enhances the efficacy of gemcitabine in suppressing the tumor growth (115). Consistent with this pre-clinical study, a recent clinical trial indicates that treatment of pancreatic cancer patients with a CCR2 antagonist (CCX872) in combination with FOLFIRINOX regimen (i.e., a combination of five chemotherapy agents) improve overall survival (102). These results suggest that elimination of macrophages via chemokine receptor antagonists in combination with direct cancer cell killing by chemotherapy is an effective therapeutic strategy to prevent malignant tumor development (Figure 4). However, macrophage blockade may not always enhance chemotherapy efficacy. In a mouse model of pancreatic cancer, treatment with a CD40 agonist increases sensitivity of the tumor to gemcitabine via depletion of fibrosis by monocytes/macrophages. Mechanistically, a CD40 agonist induces systemic release of IFNγ that prompts classical monocytes to express matrix metalloproteinase (MMP) and recruit these anti-fibrotic monocytes/macrophages to the tumor via CCL2 (116). Therefore, in such a case, blockade of TAM accumulation by CCR2 antagonists may reduce, instead of enhance, the efficacy of gemcitabine treatment. These results suggest that a certain therapeutic treatment affects features of macrophages in the tumors, and thus application of chemokine receptor antagonists to other therapeutic modalities should be carefully evaluated.

Blockade of TAM/MAM accumulation combined with immunotherapies is another attractive therapeutic strategy to prevent malignant tumor development (117). Since cytotoxic lymphocytes (CTLs) such as CD8^+^ T and NK cells can eliminate cancer cells if they exert full cytotoxicity, several strategies to utilize their tumor killing ability have been developed. These immunotherapies such as immune checkpoint inhibitors and adoptive CTL transfer have been tested in clinical trials and demonstrated significant therapeutic effects on lymphoma and some solid tumors such as melanoma and lung cancer. However, their efficacy is so far limited in a certain fraction of patients and tumor types due to tumor-cell-intrinsic mechanisms such as the impaired antigen presentation and/or tumor-cell-extrinsic mechanisms including the accumulation of immunosuppressive cells. As previously described, TAMs/MAMs are reported to suppress functions of CD8^+^ T and NK cells *in vitro* and thus considered as attractive targets to improve efficacy of immunotherapies. In a mouse model of pancreatic cancer, treatment with anti-PD1, and anti-CTLA4 antibodies in combination with TAM depletion by a CSF1 receptor antagonist (PLX3397) blocks tumor expansion more efficiently compared with a single treatment with anti-PD1/anti-CTLA4 or PLX3397 (118). It is also reported that genetic depletion of CCR2^+^ classical monocytes (i.e., TAM progenitors) enhances accumulation of adoptively transferred CD8^+^ T cells in the primary tumor, and thereby augments the therapeutic effect of the adoptive T cell transfer therapy on the tumor growth in a melanoma model (119). These results suggest that elimination of macrophages from the tumor microenvironment can improve efficacy of checkpoint inhibitors or adoptive CTL transfer. Therefore TAM/MAM blockade by chemokine receptor antagonists combined with immunotherapies can be a novel therapy for malignant tumors. However, target chemokine receptors should be carefully selected since recruitment of CD8^+^ T or NK cells in the tumor sites is also regulated by chemokine signals. It has been reported that CD8^+^ T cells utilize several chemokine receptors such as CCR4, CCR5, CCR7, CCR9, CCR10, and CXCR3 for their trafficking depending on their activation status (120). NK cells also express several chemokine receptors including CCR1, CCR2, CCR5, CCR7, CXCR1, CXCR3, CXCR4, and CXCR6 (121), suggesting that antagonists for these receptors have a potential risk to reduce the efficacy of immunotherapies. However, a recent study using a B16 mouse melanoma model demonstrated that neither *Ccr2* nor *Ccr5* deficiency affect tumor infiltration of adoptively transferred CD8^+^ T cells, despite the fact that the tumor expresses high levels of CCL2 and CCL5 (ligands for CCR2 and CCR5, respectively). In contrast, *Cxcr3* deficiency significantly reduces the recruitment of CD8^+^ T cells in the B16 tumors (122). The loss of *Cxcr3* also significantly reduces NK cell accumulation in metastatic tumors established by B16 cells (123). These results suggest that activated CD8^+^ T and NK cells may predominantly utilize CXCR3 signals for their tumor infiltration. It is thus likely that blockade of MAM-recruiting chemokine receptors such as CCR1, CCR2, CCR5 has minimum effects on the tumor infiltration of CD8^+^ T and NK cells, which is indispensable for immunotherapy efficacy. In line with this notion, the combined treatment with a CCR1 antagonist and anti-PDL1 antibody significantly reduces tumor burden compared to either of single treatments in a mouse model of breast cancer (124). It is also reported that treatment with a CCR2 antagonist in combination with anti-PD1 antibody suppresses tumor growth in a mouse model of pancreatic cancer, whereas single treatment with anti-PD1 antibody is not effective (125). These pre-clinical data suggest that blockade of macrophage-recruiting chemokine receptors combined with immunotherapy is an attractive approach. However, this combination therapy may not be effective in a certain fraction of solid tumors that do not express sufficient levels of CXCR3 ligands (CXCL9 and CXCL10) and fail to recruit tumoricidal CD8^+^ T cells (126–128). A recent study using mouse models of ovarian cancer has shown that the reduced production of CXCL9 and CXCL10 from cancer cells is caused by enhancer of zeste homolog 2 (EZH2) mediated histone modification and DNA methyltransferase 1 (DNMT1) mediated DNA methylation of the chemokine genes (129). Interestingly, this study also demonstrates that treatment of tumor-bearing mice with epigenetic modulators, i.e., combination of EZH2 and DNMT1 inhibitors, increases tumor expression of CXCL9/CXCL10 and improves therapeutic efficacy of anti-PDL1 antibody and adoptive transfer of CD8^+^ T cells by enhancing T cell migration toward tumors. Given the non-redundant requirement of CXCR3 signaling for tumoricidal T cell trafficking to the tumor (122), these epigenetic modulators can enhance efficacy of combination therapy consisting of TAM/MAM blockade and checkpoint inhibitors or CTL transfer. Another attractive approach to enhance efficacy of the combination therapy is engineering of CTLs to express receptors for chemokine ligands that are abundant in the tumor microenvironment. A recent study demonstrated that genetic engineering of CD8^+^ T cells with CCR4 enhances their migration toward CCL22 secreted from Panc02 pancreatic cancer cells *in vitro*, and that adoptive transfer of the CCR4-engineered T cells into the Panc02 tumor-bearing mice eradicate the established tumor more efficiently than the infusion of non-engineered T cells (130). It is also reported that introduction of CXCR2 in tumor antigen specific CD8^+^ T cells enhances their infiltration into the tumor that expresses the ligand CXCL1 and thereby reduces tumor growth in a mouse model of colon cancer (131). Collectively, TAM/MAM blockade by chemokine receptor antagonists in combination with immunotherapies seems to be a promising strategy to prevent the progression of solid tumors (Figure 4).

## Future perspective

Different studies have shown that accumulation of TAMs/MAMs play pivotal roles in the establishment of lethal metastatic tumors. As summarized in this review, several mouse models of metastatic tumors have identified chemokine signals that promote TAM/MAM accumulation and thus can be novel therapeutic targets to block the macrophage-promoting metastasis formation. Pre-clinical studies also suggest that TAM targeting by chemokine receptor antagonists, combined with immunotherapy has the ability to exert synergistic therapeutic effects. Further, this can be enhanced by promoting tumor infiltration of effector CTLs via chemokine signal modification. Further investigation of the synergistic effects of TAM/MAM targeting chemokine antagonists on the CTL recruitment and immunotherapy efficacy will lead to the establishment of effective therapies for metastatic disease. Since predominant chemokine signals utilized for macrophage accumulation can be changed by the tumor microenvironment, a database showing chemokine expression profiles of solid tumors with different subtypes, stages, and treatment history will be helpful to investigate the optimal combination of target chemokine receptors. Identification of environmental factors that induce macrophage-recruiting chemokines is also important since these factors can be alternative therapeutic targets. Another aspect to be considered is that tumor metastasis is supported not only by MAMs but also by other immune cell types such as T_reg_ cells and MDSCs (5). As described above, TAMs can recruit T_reg_ cells to the primary tumors via secretion of CCL20 or CCL22 (39, 40). It is also reported that T_reg_ cell recruitment to primary mammary tumors in mice is promoted by a CCL5-mediated mechanism (132). Several studies have reported that accumulation of MDSCs in the primary tumors is regulated by CXCL5, CXCL8, and CXCL12 depending on the models (133–135). However, the involvement of these chemokine signals in the accumulation T_reg_ cells and MDSCs in the metastatic site has not yet been investigated. A recent study indicates that monocytic MDSCs recruited to the pulmonary metastasis foci originate from circulating classical monocytes (44) that are recruited by the CCL2-CCR2 axis (43), which suggests a significant contribution of CCL2 to MDSC recruitment to the metastatic site. Although their roles at metastatic sites remain to be identified, T_reg_ cells, and MDSCs in the primary tumors are known to suppress CTL functions and are considered as targets to improve immunotherapy. Therefore, deciphering the chemokine signals that recruit T_reg_ cell and MDSC to metastatic tumors, as well as their correlations with MAM-recruiting chemokines will be important to determine effective chemokine receptor antagonists to combine with immunotherapies. Results from these basic studies will lead to novel therapeutic strategies, i.e., TAM/MAM blockade in combination with chemo-/immunotherapies by targeting chemokine signals. Further studies in preclinical models and patient samples are required for the clinical application of combination therapies to metastatic tumors to be realized.

## Author contributions

TK writing the manuscript and preparing the figures. TK and DA review and revision of the manuscript and figures.

### Conflict of interest statement

The authors declare that the research was conducted in the absence of any commercial or financial relationships that could be construed as a potential conflict of interest.

## Abbreviations

ATF4: activating transcription factor 4
CCL: CC-chemokine ligand
CCR: CC-chemokine receptor
CITED2: Cbp/p300-interacting transactivator with Glu/Asp-rich carboxy-terminal domain-2
CSF1: colony stimulating factor 1
CTL: cytotoxic lymphocyte
CXCL: CXC-chemokine ligand
CXCR: CXC-chemokine receptor
EGF: epidermal growth factor
EMT: epithelial mesenchymal transition
IGF1: insulin like growth factor 1
LKB1: liver kinase B1
MDSC: myeloid derived suppressor cell
MAM: metastasis-associated macrophage
mTROC1: mammalian target of rapamycin complex 1
NK cell: natural killer cell
PyMT: Polyoma Middle T oncogene
RCC: renal cell carcinoma
RKIP: Raf kinase inhibitory protein
TAM: tumor associated macrophage
TGFβ, transforming growth factor β T_reg_ cell: regulatory T cell
VEGF: vascular endothelial growth factor.
